# Physical Activity Policies for Children and Adolescents in Brazil: Analysis for the Report Card Brazil on Physical Activity for Children and Adolescents

**DOI:** 10.3390/ijerph191610152

**Published:** 2022-08-16

**Authors:** Diego Augusto Santos Silva, Carolina Fernandes da Silva

**Affiliations:** Sports Center, Physical Education Department, Federal University of Santa Catarina, Florianópolis 88040-900, SC, Brazil

**Keywords:** delivery of health care, child health, adolescent health, health policy, sedentary behavior

## Abstract

The aim of this study was to investigate physical activity (PA) policies in Brazil through current actions/programs to promote PA for children and adolescents. All 23 official websites of federal government agencies in Brazil [eighteen ministries, two secretariats (linked to ministries) and three ministry equivalent agencies] were visited. All programs/actions were analyzed according to indicators of the Global Matrix project from the Active Healthy Kids Global Alliance (AHKGA) and the Health Enhancing PA Policy Audit Tool, version 2, recommended by the World Health Organization. Furthermore, we used the analysis of “Strengths, Weaknesses, Opportunities and Threats” (SWOT) for the policies. Seventeen programs/actions included the promotion of PA for children and adolescents in Brazil, however, none of them had this as their main objective, and none were planned as a public policy action aimed at the promotion of PA. The overall score of the assessment instrument was 37 (out of a total of 100), which classifies Brazil as having a D+ grade according to AHKGA criteria. Brazil needs to define PA as a state policy so that the actions identified in this study can have positive effects on children and adolescents.

## 1. Introduction

The literature has shown the positive effects of physical activity on the physical and mental health of children and adolescents [1]. However, in 2016, the prevalence of physical inactivity was estimated as 81% in adolescents aged 11–17 years around the world, being higher in female adolescents (85.1%) and in upper-middle-income countries (83.9%) [2]. For this reason, greater dissemination of recommendations for the practice of physical activity in children and adolescents should be encouraged in different countries and in different contexts (community, school, neighborhood and family).

The socio-ecological health model highlights multiple factors that can affect people’s health (society, community, interpersonal relationships, individual factors) [3]. Since physical activity is one of the important factors for health promotion in all age groups, the socio-ecological health model can be a strategy for understanding the reasons why people do not practice physical activity on a regular basis [4]. Once these actions are understood, strategies for addressing physical inactivity can be encouraged for all age groups [4,5]. An example of these global strategies for addressing physical inactivity based on the socio-ecological health model is the Global Matrix project, led by the Active Healthy Kids Global Alliance (AHKGA), which monitors physical activity indicators (health behaviors; sources of influence; government strategies) among children and adolescents around the world [6,7].

The investigation/analysis of national physical activity policies is one of the indicators highlighted in documents from the World Health Organization, Geneva, Switzerland for physical activity promotion [8,9]. Brazil stands out in the health promotion scenario because it has a universal and free health system for the entire population [10]. This is a positive point when thinking about health care in all age groups. However, the promotion of physical activity involves professionals from different sectors of society, such as health, education, environment, infrastructure, leisure, sport and economy [8]. For this reason, national physical activity policies must consider different fields of action in society to reach greater number of individuals and promote active lifestyle for the population.

This study is part of the Report Card Brazil on Physical Activity for children and adolescents project, which is a partnership between the Federal University of Santa Catarina, Florianopolis, Brazil and the Active Healthy Kids Global Alliance [11,12]. For the first time, national physical activity policies for children and adolescents in Brazil are being described and analyzed in the light of the socio-ecological theory and based on the World Health Organization recommendations [8]. This study aims to investigate the features of the national physical activity policies in Brazil through actions and programs to promote physical activity for children and adolescents.

## 2. Materials and Methods

This study analyses policies to promote physical activity for children and adolescents in Brazil and was developed with a focus on actions at federal level. Brazil is a federative republic formed by 26 states and a Federal District. These states are grouped into five geographic regions (Midwestern, Northeastern, Northern, Southeastern and Southern), which together represent a population of ~215,000,000 inhabitants [13].

The strategy of visiting all official websites of federal government agencies in Brazil to analyze all current programs/actions, at national level, was adopted. In December 2021, Brazil had eighteen ministries, two secretariats (linked to ministries) and three ministry equivalent agencies [14]. All these 23 official websites of the Brazilian federal government were visited in December/2021, January/2022 and February/2022 to obtain information on physical activity programs/actions for children and adolescents [15,16,17,18,19,20,21,22,23,24,25,26,27,28,29,30,31].

The focus of the analysis of programs/actions is aligned with indicators recommended by the Global Matrix project of the Active Healthy Kids Global Alliance (AHKGA), which focuses on the analysis of policies to promote physical activity for children and adolescents and includes two dimensions [32]: (1) Evidence of leadership and commitment in providing physical activity opportunities for children and adolescents; (2) Allocated funds and resources for the implementation of physical activity promotion strategies and initiatives for children and adolescents, and also progress demonstrated through the key stages of public policy making (i.e., policy agenda, policy formation, policy implementation, policy evaluation and decisions about the future).

To analyze these governmental markers, the present study used the Health Enhancing Physical Activity (HEPA) Policy Audit Tool (PAT)—version 2 [8], developed by the national approaches working group of the WHO HEPA Europe network that provided a framework covering all the policy domains informed by the report card. This analysis tool was adapted to the Global Matrix—AHKGA [9] and allows for the computation of an evaluation score for the different domains. After computing the score, a grade that could vary from A+ to F in descending order of quality was assigned, according to literature recommendations [9,32].

The present study also analyzed the Strengths, Weaknesses, Opportunities and Threats (SWOT) of programs/actions to promote physical activity for children and adolescents. The SWOT strategy is used in the planning, analysis, and evaluation of government policies and actions in different areas of knowledge and allows for the synthesis of different internal (Strengths and Weaknesses) and external (Opportunities and Threats) aspects that affect government policies [33,34,35], serving as instrument for reflection, reformulation and application of these actions.

## 3. Results

There are 17 programs/actions in Brazil that in some way promote physical activity for children and adolescents. The majority of these programs/actions aim to promote sport as agent of social transformation. In addition, none of these programs/actions used behavior change theories for health promotion (Table 1).

Of the 17 existing programs/actions, only two of them (“*Academia da Saúde*” and “*Saúde na Escola*”) use the terminology “physical activity” to refer to the program/action activities. The others use the terminology “sport” or those closest to “sport” to report the proposed actions (i.e., sport, sport practice, etc.). Of the seventeen current programs/actions, one is linked to the Ministry of Health of Brazil, another program/action is governed by two Ministries (Education and Health), one is governed by the Ministry of Education of Brazil, eleven programs/actions are governed by the Ministry of Citizenship, one is governed by the Ministry of Tourism and two programs/actions by the Ministry of Defense (Table 1).

On the official website of each of the programs/actions, the objectives, the activities performed, the way in which each municipality in the country can benefit from the initiative and guidelines for the application of programs/actions that serve as guide for public managers were observed. In the 17 websites visited and in the official documents of the federal government, only four programs/actions reported the amount that the federal government invested in these programs/actions. None of the websites and documents provided information on the evaluation and/or impact of these programs/actions in the promotion of physical activity or even in the promotion of sports (Table 1).

One program/action is exclusively focused on providing physical infrastructure for municipalities that adhere to it (e.g., construction of spaces with equipment for physical activity). Such physical infrastructure is intended for the construction of suitable spaces for the implementation of the program/action. Eight programs/actions are exclusively focused on providing qualified personnel to work on activities that they develop. Four programs/actions are intended to provide physical infrastructure and qualified personnel for municipalities that adhere to them, which allows, simultaneously, the construction of favorable spaces for the implementation of the program/action and the provision of qualified personnel to work and supervise the proposed activities. The other four programs/actions provide personnel infrastructure to work and supervise the proposed activities and adequate teaching materials (Table 1).

The SWOT analysis allowed us to highlight the internal (strengths and weaknesses) and external (opportunities and threats) aspects of programs/actions to promote physical activity for children and adolescents in Brazil. In this context, ten characteristics considered as a strength and nine characteristics considered as weaknesses stood out. In addition, eight characteristics considered as opportunities and eight characteristics considered as threats stood out (Table 2).

According to methodology developed by WHO and adapted by AHKGA for the evaluation of public policies on physical activity, the overall score of Brazil for programs/actions to promote physical activity for children and adolescents was 37 (out of a total of 100), which classifies Brazil as having a D+ grade. The most negative aspect of these programs/actions was the “Monitoring and evaluation plan” dimension. On the other hand, these programs/actions stood out positively in the “Identified accountable organization(s)” dimension (Table 3).

## 4. Discussion

The fact that Brazil has programs focused on promoting sport is positive because the practice of sport is one of the means of increasing the levels of physical activity in the pediatric population [36,37]. Involvement in sports should be encouraged at all stages of childhood, as this is an educational way for young people to move and interact socially [36,37]. However, this strategy should not be the only one used for behavior change. The actions developed in Brazil focus on sport as agent of social transformation, which is a positive fact, but there is no program/action that focuses on increasing levels of physical activity and changing the behavior of children and adolescents. The lack of specific programs/actions may be one of the reasons why physical inactivity and sedentary behavior are highly prevalent among young Brazilians [38,39] and why physical inactivity is responsible for the high burden of morbidity and mortality in the Brazilian population of different age groups [40,41,42].

One of the results that this research found was that only in four programs/actions, the financial resources allocated to projects were made explicit [17,20,30,31]. Knowledge of the amount of resources allocated to a public policy is useful for analyzing the cost-effectiveness of the program and its efficiency in reaching the population [43]. Since 2016, Brazil has been suffering from a decrease in public health resources [10]. The Constitutional Amendment No. 95/2016 limited from 2017, the expansion of public spending for the next 20 years in Brazil [10]. Many studies have reported that this austerity measure will lead to the disruption of the Brazilian healthcare system, which is free and universal, and will focus on the healthcare privatization in Brazil [44]. Interestingly, six programs/actions found in the present study were created after 2017 [23,24,25,27,29,30], which may represent an advance for the promotion of physical activity for children and adolescents; however, of these six programs/actions, only one of them [30] makes it clear on its website the amount of resources that have already been allocated to this action. The lack of information is limiting for the evaluation of the program/action and does not allow, for example, the calculation of how much this resource allocated to the program represents for each Brazilian child and adolescent.

The evaluation of programs/actions is a stage in the development cycle of public policies with regard to the management and planning of actions. It is understood as a constitutive part of the public policy process and is linked to the maintenance of the quality of services offered by the government, and also serves to regulate actions, omissions, decisions and non-decisions on each program/action [45]. Of programs/actions found in the present study, none of them presented information about the monitoring system and the evaluation plan of the impact and effectiveness of proposed activities. This can be considered a limitation of physical activity programs aimed at children and adolescents, because without a frequent evaluation plan, there is no way to reprogram the objectives and scope of actions. There is a theoretical explanation for the lack of an evaluation plan in Brazilian policies. The main one is that until the end of the 1980s, the country lived under the tutelage of an authoritarian, non-democratic government, averse to submitting policies to evaluation or any other type of analysis [45]. Although more than 30 years have elapsed since the end of the Military Dictatorship in Brazil, the change in mentality from authoritarian regimen to participatory regimen is slow and sometimes still presents some setbacks that require active actions by civil society. In addition, in the authoritarian Brazilian government, policies were focused and residual, far from the concept of a welfare state present in Europe and North America in the 80s and 90s, which were a stimulus for the development of evaluation actions [45]. In this sense, to promote physical activity policies for children and adolescents, Brazil needs to change the mentality of promoting policies and focusing on intersectoral actions, with the active participation of society.

An opportunity that can be highlighted in this study is that all 17 programs/actions have high potential for reaching the Brazilian population, mainly because they are actions that focus on sports, cultural and leisure activities that have great appeal for society. In addition, these actions are free, which for a country such as Brazil with huge inequalities, is relevant for society. The topic of physical activity is constantly debated in the media and the launch of the Physical Activity Guide for the Brazilian Population in 2021 [46] is an opportunity to discuss this topic in schools, community and families.

According to the AHKGA methodology for evaluating physical activity policies [9], the present study reported score of 37 (out of a total of 100) in the Health Enhancing Physical Activity Policy Audit Tool. The higher the score in this tool, the more appropriate the country is in terms of national physical activity policies [9]. In Wales, a score of 54 (out of a total of 100) was reported for physical activity policies for children and adolescents in the year 2018 [9]. As in Brazil, in Wales, the most fragile dimension of physical activity policies was the “Monitoring and evaluation plan”, which reveals that monitoring and evaluating physical activity actions is necessary and is rarely carried out by national governments. In the search for this article, only the study with data from Wales [9] has used the same tool to compute scores for PA policies.

This study has some limitations that need to be reported. First, a major limitation of this study is the reliance only on what is published on the policy websites in order to evaluate the programs. The financing and evaluation actions of the programs can be conducted internally to the program and, as such information is not on the program’s website, it was not possible to analyze it. However, Brazilian legislation reinforces the need for transparency of information on public resources and public actions. Thus, this study suggests as a guideline for Brazilian ministries involved in the programs/actions that all information on allocated resources and evaluation of actions be publicized on the website. Second, the governmental actions analyzed in this study concern the policies of the Brazilian federal government. Brazil has approximately 5000 municipalities [13] and these locations can also propose programs/actions, independent of the federal government. In any case, Brazilian municipalities adhere to federal government policies; thus, it is believed that the programs/actions investigated in this article show the reality of the country. Third, the SWOT analysis was described based on the expertise of the group of researchers who worked in this study. This could result in differences if we opted for the analysis of another work group; however, the strategy used in this study is in agreement with other studies that used the same SWOT technique [33,34,35]. The use of the HEPA PAT, version 2 [8] for the analysis of programs/actions is strength of this study and complemented the SWOT analysis. Finally, many of these programs/actions were created in the pre-COVID-19 pandemic period and may have been harmed due to the change in social dynamics caused by the pandemic such as social distancing, the allocation of public resources to the pandemic situation and the suspension of face-to-face activities of many programs/actions. Thus, it is expected that in future editions of the Report Card on Physical Activity for Brazilian children and adolescents, the evaluation of these programs/actions can continue to demonstrate the impact of the COVID-19 pandemic on national physical activity policies for the Brazilian pediatric population.

## 5. Conclusions

It could be concluded that Brazil has many programs/actions at federal level that in some way promote physical activity for children and adolescents. However, none of these programs/actions have the promotion of physical activity as their main objective and few programs/actions have reported the amount of resources allocated to these actions on the websites.

Brazil has potential in human resources to develop and direct the programs/actions identified in this study to promote physical activity for children and adolescents in the school, community and/or family environment. For this, it is necessary to define as a government policy the promotion of physical activity for the pediatric population and the use of sports and other actions as a means of making children and adolescents healthier.

## Figures and Tables

**Table 1:** Programs/Actions for the promotion of physical activity for children and adolescents in Brazil.

Programs/Actions	Creation Year	Ministry	Main Objective	Target Population	Dimension	Investment
Academia da Saúde [15]	2011	Health	Contribute to the promotion of health and production of care and healthy lifestyles for the population through the implementation of centers with infrastructure and qualified professionals	The entire population	Physical and personnel infrastructure	Not informed
Saúde na Escola [16]	2007	Education;Health	Develop health promotion actions articulated between the health and education sectors, aiming at comprehensive care and education to improve the health of the school population	Basic Education students, education and health managers and professionals, the school community and, more broadly, students from the Federal Network of Professional and Technological Education and Youth and Adult Education	Physical and personnel infrastructure	Not informed
Novo Mais Educação [17]	2016	Education	Improve learning in Portuguese and Mathematics in elementary school, by extending the school day of children and adolescents. The Program has been implemented through pedagogical monitoring in Portuguese and Mathematics and the development of activities in the fields of arts, culture, sports and leisure	Children, teenagers and adults	Personnel infrastructure and adequate teaching material	R$277,937,003.00 *
Segundo Tempo [18]	2003	Citizenship	Democratize access to the practice and culture of educational sports, promoting the integral development of children and adolescents as a factor in forming citizenship and improving the quality of life, primarily for those living in areas of social vulnerability and who are preferably enrolled in the public school system	Children, adolescents and university students (including people with disabilities)	Personnel infrastructure and adequate teaching material	Not informed
Esporte e Lazer da Cidade [19]	2011	Citizenship	Democratize access to leisure and recreational sport, favoring underprivileged communities	Children, adolescents, young people, adults and the elderly (including people with disabilities)	Personnel infrastructure	Not informed
Forças no Esporte [20]	2003	Defense	Democratize access to the practice and culture of sports and promote the integral development of children and adolescents, offering educational sports activities, leisure and complementary activities	Children and adolescents	Physical and personnel infrastructure	R$20,600,000.00
Projeto João do Pulo [21]	2015	Defense	Democratize access to the practice and culture of sports and promote the integral development of children and adolescents, offering educational sports activities, leisure and complementary activities	Children and adolescents with disabilities	Physical and personnel infrastructure	Not informed
Luta pela Cidadania [22]	2016	Citizenship	Offer the practice of martial arts that encourage the integral development of children, adolescents and adults	Children, teenagers and adults	Personnel infrastructure and adequate teaching material	Not informed
Brincando com Esporte [23]	2018	Citizenship	Give children and adolescents, during the two annual school vacation periods, options for sports and leisure to fill their free time	Children and adolescents (including people with disabilities)	Personnel infrastructure and adequate teaching material	Not informed
Comunidades ribeirinhas da Amazônia [24]	2019	Citizenship	Develop sports, fighting sports and martial arts, cultural and leisure practices with riverside communities in the Amazon	Communities of people living on the banks of rivers who have artisanal fishing as their main survival activity	Personnel infrastructure	Not informed
DELAS [25]	2019	Citizenship	Contribute to strengthening female empowerment, by offering fighting sports and martial arts activities and promoting a cycle of debates on rights, forms of violence and the contexts in which they can be developed	Women, from 12 years of age.	Personnel infrastructure	Not informed
Esporte e Cidadania [26]	2016	Citizenship	Democratize access to sport for children, adolescents and young people	Children, adolescents and young people, aged 6–21 years in a situation of social vulnerability and/or who comply with socio-educational measures in the internment and/or semi-liberty condition	Personnel infrastructure	Not informed
Virando o Jogo [27]	2019	Citizenship	Provide access to the practice and culture of sports and leisure, with an emphasis on the development of martial arts activities	Children, adolescents, young people, adults, the elderly and people with disabilities	Personnel infrastructure	Not informed
Aldeia Viva [28]	2017	Citizenship	Encourage, value and strengthen sports and leisure practices in indigenous communities	Indigenous children, adolescents and adults.	Personnel infrastructure	Not informed
Iniciação e aprimoramento de modalidade esportiva [29]	2019	Citizenship	Expand the possibilities of access and improvement of skills in sports, offering sports, with educational, inclusive and recreational nature	Children and adolescents (including those with disabilities)	Personnel infrastructure	Not informed
Seleções do Futuro [30]	2019	Citizenship	Encourage, develop and democratize access to sports training in the soccer modality	Children and adolescents	Personnel infrastructure	R$6,420,381.55
Pracinhas da Cultura [31]	2011	Tourism	Integrate cultural programs and actions, sports and leisure practices, training and qualification for the job market, social assistance services, violence prevention and digital inclusion policies in the same space	The entire population	Physical infrastructure	R$729,066,324.37

SUS: Unified Health System; Investment disclosed on the action website in December/2021; * Total value calculated by adding the years 2017, 2018 and 2019; R$: Real (Brazilian Currency); 1 US$ = R$5.50 (in February/2022).

**Table 2:** Analysis of Strengths, Weaknesses, Opportunities and Threats (SWOT) of programs/actions to promote physical activity for children and adolescents in Brazil.

**STRENGTHS**	**WEAKNESSES**
Practice of organized sports	Lack of at least one program/action with main focus on promoting physical activity
Promotion of physical activity even in a secondary way	National bureaucratization for the implementation of programs/actions
Use of sport as agent of social transformation	Difficulty in defining regionalized goals
Democratize access for the practice and culture of sports	Efforts to register municipalities in programs/actions
Actions available for the entire national territory	Lack of studies on the impact of programs/actions
Users access programs/actions for free	Lack of studies on the evaluation of programs/actions
Provision of qualified personnel to supervise actions	Lack of studies on the effectiveness of programs/actions
Provision of physical infrastructure for the development of actions	Lack of information on investment in each program/action
Supply of didactic material for the development of actions	Lack of information on the number of individuals benefitted from each of the programs/actions
Involvement of several Brazilian government ministries	
**OPPORTUNITIES**	**THREATS**
The topic of physical activity is constantly in evidence in the media	Lack of public policy to promote physical activity in Brazil
Several actions can be carried out in the school environment	Lack of the topic physical activity in the permanent agenda of the federal government
Several actions can be carried out in the neighborhood	Lack of dialogue between universities and strategic sectors of the federal government
Several actions can be carried out with the support of friends and family	Decrease in financial resources for scientific research
Great interest of the population in accessing programs/actions as it is a benefit without financial compensation	Increase in political and social tensions in Brazil
Brazil has a tradition of practicing sports in and out of the school environment	Increase in social and regional inequalities in Brazil
The COVID-19 pandemic demonstrated the need for the regular practice of physical activities in all age groups	Lack of transparency in the allocation of financial resources for the implementation of programs/actions
The launch in 2021 of the Physical Activity Guide for the Brazilian population	The global uncertainty in the face of the COVID-19 pandemic

**Table 3:** Description of the Health Enhancing Physical Activity (HEPA) Policy Audit Tool (PAT) score for physical activity programs/actions in Brazil.

Criteria	Narrative	Score
Number and breadth of relevant policies	There are 17 programs/actions in Brazil that at some level promote physical activity for children and adolescents. Such programs/actions are linked to the areas of Education, Health, Citizenship, Leisure and Sport	6/10
Identified supporting actions	None of these programs/actions have the promotion of physical activity as their main objective and none of them were planned as a public policy action aimed at promoting physical activity for children and adolescents. Only two of them use the terminology “physical activity” to refer to program/action activities. The others use the terminology “sport” or those closest to “sport” to report the proposed actions (i.e., sport, sport practice, etc.)	2/20
Identified accountable organization(s)	In all programs/actions, it is possible to identify the Ministry of Brazil responsible for activities	25/25
Identifiable reporting structures	None of programs/actions provide annual technical reports on the actions carried out and none of programs/actions provide annual strategic reports on activities to be carried out.	0/15
Identified funding/resourcing (national programs)	Only four of the seventeen programs/actions report the amount invested in activities on their websites/documents	4/20
Monitoring and evaluation plan	None of programs/actions present information about the monitoring system and the evaluation plan of the impact and effectiveness of proposed activities	0/10
Overall grade: 37 (D+)		

## Data Availability

Not applicable.
